# Loss of cholinergic innervation differentially affects eNOS-mediated blood flow, drainage of Aβ and cerebral amyloid angiopathy in the cortex and hippocampus of adult mice

**DOI:** 10.1186/s40478-020-01108-z

**Published:** 2021-01-07

**Authors:** Shereen Nizari, Jack A. Wells, Roxana O. Carare, Ignacio A. Romero, Cheryl A. Hawkes

**Affiliations:** 1 School of Life, Health and Chemical Sciences, Open University, Milton Keynes, UK; 2 Centre for Advanced Bioimaging, UCL, London, UK; 3 Clinical and Experimental Sciences, University of Southampton, Southampton, UK; 4 Department of Biomedical and Life Sciences, Lancaster University, Lancaster, UK

**Keywords:** Alzheimer’s disease, Cholinergic, Clearance, eNOS, Vascular reactivity

## Abstract

Vascular dysregulation and cholinergic basal forebrain degeneration are both early pathological events in the development of Alzheimer’s disease (AD). Acetylcholine contributes to localised arterial dilatation and increased cerebral blood flow (CBF) during neurovascular coupling via activation of endothelial nitric oxide synthase (eNOS). Decreased vascular reactivity is suggested to contribute to impaired clearance of β-amyloid (Aβ) along intramural periarterial drainage (IPAD) pathways of the brain, leading to the development of cerebral amyloid angiopathy (CAA). However, the possible relationship between loss of cholinergic innervation, impaired vasoreactivity and reduced clearance of Aβ from the brain has not been previously investigated. In the present study, intracerebroventricular administration of mu-saporin resulted in significant death of cholinergic neurons and fibres in the medial septum, cortex and hippocampus of C57BL/6 mice. Arterial spin labelling MRI revealed a loss of CBF response to stimulation of eNOS by the Rho-kinase inhibitor fasudil hydrochloride in the cortex of denervated mice. By contrast, the hippocampus remained responsive to drug treatment, in association with altered eNOS expression. Fasudil hydrochloride significantly increased IPAD in the hippocampus of both control and saporin-treated mice, while increased clearance from the cortex was only observed in control animals. Administration of mu-saporin in the TetOAPPSweInd mouse model of AD was associated with a significant and selective increase in Aβ40-positive CAA. These findings support the importance of the interrelationship between cholinergic innervation and vascular function in the aetiology and/or progression of CAA and suggest that combined eNOS/cholinergic therapies may improve the efficiency of Aβ removal from the brain and reduce its deposition as CAA.

## Introduction

Increasing evidence suggests that structural and functional alterations of the cerebrovasculature contribute to the aetiology and/or progression of Alzheimer’s disease (AD). In fact, vascular pathology has been suggested to be one of the earliest indicators of the development of AD [38, 39] and differential perfusion of AD-sensitive brain areas such as the hippocampus, frontal and temporal lobes are present in people both with mild cognitive impairment and dementia [2, 18, 34].

Cerebral amyloid angiopathy (CAA) is the most common form of cerebrovascular pathology in AD [42] and is characterised by the deposition of β-amyloid (Aβ) peptides in the walls of cerebral arteries and capillaries [90]. While parenchymal plaques are made up predominantly of Aβ42, Aβ40 is more commonly observed in CAA. CAA develops topographically, presenting initially in the occipital lobe, followed by the temporal, frontal and parietal lobes, then in the hippocampus and entorhinal cortex at later stages [5, 83, 84]. In addition to causing dysfunction and death of mural and endothelial cells, recent studies suggest that CAA contributes to impaired hemodynamic responses in both individuals with AD and people with hereditary CAA [4, 63, 77, 87, 94].

A key pathological feature of sporadic CAA is a failure of clearance of Aβ from the brain, which is mediated via enzymatic degradation, uptake in microglia and astrocytes and transcytosis across the blood–brain barrier [54, 55, 93]. Aβ is also removed from the brain along the walls of the capillaries and arteries via intramural periarterial drainage (IPAD) and/or glymphatic drainage [7, 36, 56].

The IPAD hypothesis of Aβ clearance is based in part on experimental observations that nanoparticles, solutes and Aβ injected into the interstitial fluid (ISF) of deep brain structures are transported along and localise to cerebrovascular basement membranes (CVBM) in cortical and leptomeningeal vessels [3, 13, 27–29]. In the mouse brain, this process occurs very rapidly, within 5–10 min of injection [8, 13, 27–29]. Since the pattern of distribution of solutes closely mimics that of Aβ accumulation in CAA and other angiopathies [14, 41, 85], failure of IPAD is a key element of CAA pathology. However, as the movement of solutes along CVBMs is counter to the direction of blood flow, the driving force that underlies IPAD is still unknown. Recent mathematical modeling suggests that oscillating pulsatile flow generated by the focal contraction and relaxation of arteries drives IPAD [1] and this is supported by recent experimental data [3, 64, 88]. Localised arterial dilatation and contraction can occur both spontaneously (e.g. vasomotion) and in response to neuronal activity (e.g. neurovascular coupling, NVC) and both mechanisms have been shown to be decreased in AD [19, 69, 79].

Smooth muscle cells that regulate arterial contraction and contribute to the regulation of cerebral blood flow (CBF) in the cortex and hippocampus receive innervation from cholinergic neurons that originate in the basal forebrain. Release of acetylcholine (ACh) via stimulation of the basal forebrain or increasing cholinergic tone using acetylcholinesterase inhibitors (AChEIs) has been shown to increase CBF in the cortex and hippocampus [50, 72]. ACh induces vasodilation primarily by stimulating the production of nitric oxide (NO) via activation of endothelial nitric oxide synthase (eNOS) [23, 97], although stimulation of neuronal NOS (nNOS)-containing interneurons can also increase CBF [15, 89]. ACh-induced activation of eNOS is mediated principally by binding to muscarinic receptors that stimulate calcium release and binding of calcium-calmodulin to eNOS [25]. ACh activation of eNOS can also occur via the insulin-receptor substrate/PI3K/Akt pathway [96] and stimulation of the PI3K/Akt/eNOS pathway by the selective Rho- associated, coiled-coil containing protein kinase (ROCK) inhibitor fasudil hydrochloride, has been shown to increase CBF in mice and humans [59, 68]. Although multiple downstream signalling pathways are regulated by ROCK activity [45, 76], several studies have reported no effect of fasudil hydrochloride on cerebral haemodynamics in eNOS^−/−^ mice, suggesting that eNOS is the principal NOS isoform targeted by fasudil hydrochloride [68, 78].

Decreased expression of eNOS has been reported in the occipital cortex in AD, an area of the brain that is hypoperfused in AD [12]. Conversely, eNOS and inducible NOS (iNOS) activity have been shown to be significantly increased in the temporal and frontal cortices of AD patients [20], in association with hyperperfusion of those areas [34]. Several recent studies have reported that endogenous CAA load is increased in eNOS-deficient mice in the absence of alterations in parenchymal Aβ or increased Aβ production [6, 81], suggesting that dysfunction of eNOS may also contribute to the aetiology of CAA and that this may be related to impairments in Aβ clearance from the brain.

Loss of cholinergic neurons as an early pathological feature of AD has been known since the 1980s and underpins the rationale for the current clinical use of AChEIs for the treatment of AD [9, 22, 66]. Two recent findings from the Alzheimer’s Disease Neuroimaging Initiative have reported that vascular dysregulation is an early predictor of the progression to AD and that loss of volume in the basal forebrain precedes pathological changes in the entorhinal cortex of individuals who went on to develop AD [38, 73]. These findings suggest that the interplay between loss of cholinergic innervation and vascular dysfunction may be important in the aetiology of AD. However, although some pathological studies have examined cholinergic loss at the neurovascular unit under experimental conditions [61] and in AD [60], less has been done to directly investigate the functional outcome of perivascular cholinergic denervation. The aim of this study was to test the hypothesis that loss of cholinergic innervation decreases CBF and IPAD of Aβ from the cortex and hippocampus of wildtype mice, leading to increased CAA in the TetOAPPSweInd model of AD.

## Materials and methods

### Animals

C57BL/6 mice were bred at the Open University (OU, Milton Keynes, UK) and the University of Southampton (Southampton, UK). TetOAPPSweInd mice developed by Dr Joanna Jankowsky (Baylor College of Medicine, Texas, US) [40] were a generous gift from Dr JoAnne McLaurin (Sunnybrook Research Centre, Toronto, Canada) and were also bred on a C57BL/6 background. Food and water were provided ad libitum. All animal work was approved by the Animal Welfare and Ethics Research Boards (AWERB) at the OU, University of Southampton and UCL in accordance with Home Office regulations and project licences (PPL 70/8507 and PPL 30/3095) under the Animals (Scientific Procedures) Act 1986.

### Mu-Saporin administration

8–10 week old male C57BL/6 mice and 4-month old male and female TetOAPPSweInd mice were used for saporin injections. Mice were anesthetised under isoflurane gas and placed into a stereotaxic frame (Kopf instruments, CA, USA). Analgesia was administered intraperitoneally (Carprieve, 5% w/v, 0.32 ml/kg, Norbrook, Northamptonshire, UK) and a topical anaesthetic (Cryogesic (ethyl chloride), Acorus Therapeutics Ltd, Chester, UK) was applied before making a midline incision. 0.5 µL of mu-saporin (0.596 µg/µl, Advanced Targeting Systems, CA, USA) or 0.9% sterile saline was injected into the left and right lateral ventricles (coordinates from Bregma: AP = − 0.4 mm, ML = ∓ 1.0 mm, DV = − 2.3 mm) using a 33 gauge Hamilton syringe. Mice were able to self-administer sugar free jelly containing Carprofen (250 µg in 500 µl jelly, Zoetis, London, UK) for 1 week post-surgery. Animals were randomly assigned to receive either saline or saporin and all experimenters were blinded to treatment until statistical analysis.

### Immunohistochemistry

45 days after surgery, mice were deeply anesthetised and perfused intracardially with 0.01 M phosphate buffered saline (PBS) followed by 4% paraformaldehyde (PFA). Brains were post-fixed in 4% PFA overnight, sectioned (20 µm thickness) using a cryostat and stored at − 20 °C. Details of primary and secondary antibodies used for immunohistochemistry are listed in Additional file 1: Table 1.

For enzyme-linked immunohistochemistry, sections were washed in 0.01 M PBS, incubated with 3% hydrogen peroxide, rinsed in PBS and treated with 70% formic acid for 45 s. Sections were then blocked in 15% normal donkey or goat serum (NDS, Sigma-Aldrich, Dorset, UK), followed by incubation overnight at 4 °C with anti-Aβ40 (1:100) or anti-Aβ42 (1:100). The next day, sections were incubated with biotinylated anti-rabbit (1:400) and developed using glucose oxidase enhancement with DAB as chromagen (Sigma-Aldrich, Dorset, UK). The specificity of the anti-Aβ40 and anti-Aβ42 antibodies was verified by pre-absorbing purified human Aβ40 peptide with the anti-Aβ40 antibody (10:1 molar ratio) alone or in combination with the anti-Aβ42 antibody for 1.5 h at RT before proceeding with tissue incubation and development as described above (Additional file 2: Fig. 1a–d). Photomicrographs were obtained using a Nikon Eclipse 80i light microscope (Nikon UK Limited, Surrey, UK) and images from the hippocampus and cortex (n = 6 control and n = 7 saporin) were analysed using Fiji (NIH, Maryland, USA).

For single labelling immunofluorescence, sections were washed in 0.01 M PBS, blocked with serum and incubated overnight at 4 °C with anti-choline acetyltransferase (ChAT; 1:75), anti-p75NTR (1:350), anti-GFAP (1:500), anti-Iba1 (1:500) or anti-laminin (1:350). Sections were incubated with the appropriate fluorophore-conjugated secondary antibodies and coverslipped using Mowiol^®^ (Sigma, Dorset, UK) containing 0.1% v/v Citifluor (Citifluor ltd, London, UK).

For multiple labelling fluorescent immunohistochemistry, sections underwent antigen retrieval (Additional file 1: Table 1) and were then incubated overnight at 4 °C with either i) anti-ChAT (1:75) and anti-p75NTR (1:400), or ii) with anti-NOS (1:200) or anti-eNOS (1:200) in combination with anti-GFAP (1:2000) or anti-Iba1 (1:500). Sections were then incubated for 2 h at RT with the appropriate fluorophore-conjugated secondary antibodies, washed in PBS and coverslipped as above. The specificity of the fluorescently-conjugated secondary antibodies was verified by omitting the primary antibodies (Additional file 2: Fig. 1e–h).

For all fluorescent imaging, photomicrographs were obtained using a Leica SP5 confocal microscope using the same gain and intensity and maximum projection images were exported to Adobe Photoshop 2020 or Fiji.

### Quantification of immunohistochemistry

The density of ChAT staining (neuronal cell bodies or fibres) in each brain region was quantified from low magnification images by calculating the percentage area covered by staining using the “Analyze particle” function in Fiji (NIH. Maryland, USA). For quantification of staining in the hippocampus, overlapping images were stitched together and values from both the ipsilateral and contralateral hemispheres were averaged for each animal. For quantification of cortical images, six random non-overlapping images spanning the somatosensory cortex of the ipsilateral cortex to the somatosensory cortex of the contralateral cortex were captured and averaged per animal. A single low magnification image/animal was used to quantify ChAT staining in the medial septum. The percentage area containing microglia, astrocytes and blood vessels was also calculated using the ‘Analyze particle” function in Fiji. Additionally, for anti-laminin staining, in order to quantify density by vessel type, a mask was set to select capillaries (0–100 µm^2^) or large-diameter vessels (101 µm^2^-infinity) using images calibrated according to the scale bar. The degree of colocalization between ChAT and p75NTR and between e/NOS and laminin was determined from three images/region/animal taken at × 40 magnification using Pearson’s correlation coefficient (PCC, Coloc 2 plugin) in Fiji. For quantification of Aβ staining, images from the hippocampus and cortex taken at × 4 magnification were stitched together, images were converted to 8 bit greyscale images and the % area covered by CAA and plaques were quantified separately in Fiji.

### Arterial spin labelling MRI

Approximately 5 weeks after ICV injection, C57BL/6 mice that received saline (n = 7) or mu-saporin (n = 7) were transported to UCL Centre for Advanced Imaging and allowed to acclimate for 1 week before imaging. A 9.4 T VNMRS horizontal bore scanner (Agilent Inc., Santa Clara, CA, USA) with a 72 mm inner diameter volume coil and 2 channel array head coil (Rapid Biomedical, Columbus, OH, USA) was used for radio frequency transmission and signal detection. Mice were initially anaesthetised under 2% isoflurane in medical air and maintained under 1.5% during imaging. A rectal probe and a pressure pad (SA Instruments, Stony Brook, NY, USA) were used to measure core temperature and monitor respiration throughout the procedure. Heated water tubing and a warm air blower using a feedback system (SA Instruments, Stony Brook, NY, USA) was used to regulate the temperature of the mice to 37 °C. Following a 5 min acquisition of baseline CBF, mice were administered 10 mg/kg Fasudil hydrochloride i.p. (Tokyo Chemical Industries, Tokyo, Japan) and re-imaged 10 min later for an additional 5 min. At the end of the imaging experiments, mice were perfusion fixed with 4% PFA and their brains collected for immunohistochemistry. A total of 15 brain image slices were acquired with a thickness of 1 mm and an ‘in-plane’ resolution of 0.28 mm per mouse per experiment. Statistical Parametric Mapping (SPM, http://www.fil.ion.ucl.ac.uk/spm/) was applied to perfusion-weighted acquired ASL images [91]. Acquired images were processed using a Matlab (Mathworks, MA, USA) customised script. Regions of interest (cortex and hippocampus) were then manually traced on a single slice and quantified using a Matlab script which converted pixel intensity into CBF (ml/100 g/min) [91].

### Western blotting

45 days post-injection with saline (n = 7) or mu-p75-saporin (n = 6), C57BL/6 mice were given an overdose of sodium pentobarbitone and perfused with 0.01 M PBS. Brains were removed, dissected for hippocampus and cortex, snap frozen and stored at − 80 °C until use. Tissues were homogenised in RIPA lysis buffer (20 mM Tris pH 8.0, 0.15 M NaCl, 1.27 mM EDTA, 1 ml Igepal, 0.1% SDS, 50 mM NaF, 1.48 mM NaVO_3_ containing 1:100 Protease inhibitor cocktail [Merck Millipore, UK]), centrifuged at 10,000 g at 4 °C for 10 min and the supernatant was collected. 30 μg or 40 μg of proteins were separated by gel electrophoresis and membranes were then blocked in 8% non-fat milk before incubation with anti-eNOS (1:5000, Cell Signalling Technology, London, UK) or anti-nNOS (1:250, Cell Signalling Technology) overnight at 4 °C. Membranes were then washed in TBST before being incubated in HRP-conjugated anti-rabbbit (1:5000, Fisher Scientific) for 1 h at room temperature and developed using an enhanced chemiluminescence kit (GE Healthcare, Little Chalfont, UK). Membranes were then stripped and re-probed with anti-GAPDH (1:50,000, Sigma-Aldrich) to ensure equal protein loading. Optical density of the bands was quantified and normalised to GAPDH levels using Fiji.

### Assessment of IPAD

45 days after injection with saline or saporin, mice were anesthetised with isoflurane and placed into a stereotaxic frame. For hippocampal injections, 0.5 µL of 50 µM human Aβ40 HiLyte Fluor™ 555 (AnaSpec, California, USA) was injected into the left hippocampus (co-ordinates from Bregma: AP = − 1.9 mm, ML = 1.5 mm, DV = − 1.7 mm, n = 16 control and n = 14 saporin). Mice were perfused with PBS and 4% PFA 5 min post-injection. For cortical injections, control (n = 8) and saporin (n = 7) mice were injected with 0.25 µL of 50 µM Aβ40 HiLyte Fluor™ 555 into the right cortex (co-ordinates from Bregma AP = − 2 mm, ML = − 1.5 mm, DV = − 0.5 mm) and mice were perfused 2.5 min later. All injections were carried out at a rate of 0.2 µL/min using a 33 gauge Hamilton syringe and the injection needle was left in situ for 2 min to avoid reflux. A separate group of control and saporin-treated mice (n = 5/group) were administered fasudil hydrochloride (10 mg/kg, i.p.) 10 min before intracerebral injections. Tissue sections were processed for double-labeling immunohistochemistry as described above using anti-laminin (1:350) and anti-α smooth muscle actin conjugated to FITC (1:350; Additional file 1: Table 1). Brain sections that were ≥ 400 μm away from the site of injection were imaged for quantification. The number of capillaries, arteries and veins that contained Aβ40 HiLyte Fluor™ 555 within each image were counted manually and divided by the total area analysed, as described previously [27, 29, 61].

### Statistical analysis

Data were tested for normality using the Shapiro–Wilk test and the ROUT test was used to identify and remove statistical outliers. Comparisons between control and saporin-treated mice were analysed using two-tailed Student’s *t* test or Mann–Whitney U test where data were not normally distributed. Analysis of baseline vs stimulated CBF was carried out using paired one-tailed t-test and Wilcoxon matched-pairs signed rank test. Differences in NOS activity were analysed using one-way ANOVA with Sidak post hoc test. Differences in counts of Aβ40-positive vessels within each brain region were analysed using a one-way ANOVA with Sidak post hoc analysis or Kruskal–Wallis test with Dunn’s post hoc. In all cases, significance was set at *p *< 0.05 and data are displayed as mean ± SEM.

## Results

### mu-Saporin induces loss of cholinergic neurons and fibres

**Fig. 1:**
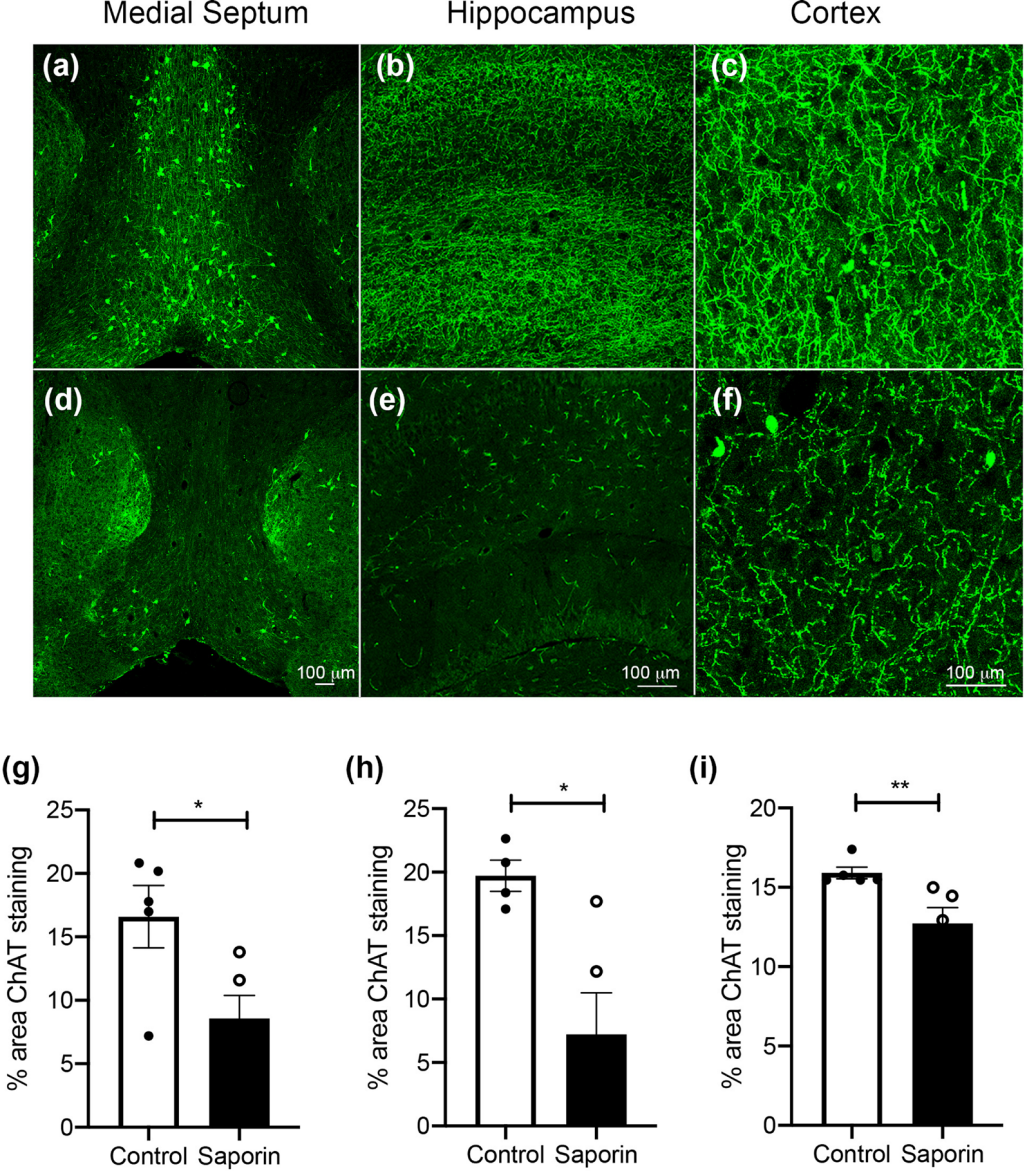
Saporin administration kills cholinergic neurons and fibres in wildtype mice. **a–f** Photomicrographs of ChAT staining in the medial septum and diagonal band of Broca (**a** and **d**), hippocampus (**b** and **e**) and cortex (**c** and **f**) in control (**a**–**c**) and mu-saporin treated C57BL/6 mice (**d**–**f**). (**g-i),** Quantification of % area covered by ChAT-positive neurons in the medial septum (**g**) and fibres in the hippocampus (**h**) and cortex (**i**). Scale bar = 100 μm. n = 5/group, *p < 0.05, **p < 0.01

In control mice, immunohistochemistry for ChAT labelled neurons in the medial septum (MS), diagonal band of Broca (DBB) and striatum (Fig. 1a). ChAT-positive fibres in the hippocampus and cortex were also observed in these animals (Fig. 1b, c). Colocalization was noted between ChAT and p75NTR in the majority of basal forebrain neurons (PCC = 0.68) as well as in fibre projections in the hippocampus and cortex (PCC = 0.25 and 0.24, respectively) in control animals (Additional file 3: Fig. 2). Administration of mu-saporin induced a significant loss of ChAT-positive, p75NTR-positive neurons in the MS and DBB (Fig. 1d, g, Additional file 3: Fig. 2), as well as fibres in the hippocampus (Fig. 1e, h, Additional file 3: Fig. 2) and cortex (Fig. 1f, i, Additional file 3: Fig. 2), confirming the usefulness of the model to induce significant death of basal forebrain cholinergic neurons and their projection fibres.

### Cholinergic loss decreases eNOS-mediated cerebral blood flow in the cortex but not the hippocampus

**Fig. 2:**
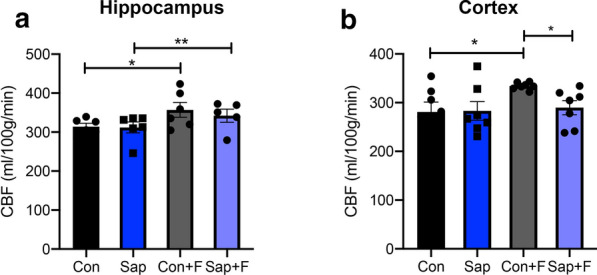
The hippocampus, but not cortex, of denervated mice remains responsive to eNOS-stimulated increase in CBF. **a** and **b** Quantification of cerebral blood flow (CBF) in the hippocampus (**a**) and cortex (**b**) of control (con) and saporin-treated mice (sap) at baseline and 10 min after administration of fasudil hydrochloride (+F), averaged over 5 min. n = 5–7/group, *p < 0.05

We have previously found that mu-saporin causes loss of cholinergic innervation of cerebral blood vessels and that this denervation is more pronounced in the cortex than the hippocampus [61]. To assess the effect of this loss on baseline and evoked CBF, arterial spin labelling MRI was used to image cerebral perfusion in the hippocampus and cortex of control and saporin-treated mice. Baseline CBF did not differ between control and saporin mice in either the hippocampus or cortex (Fig. 2a, b). Administration of fasudil hydrochloride caused a significant increase in hippocampal CBF relative to baseline in both control and saporin-treated mice (Fig. 2a). The degree of CBF increase was similar between treatment groups (Fig. 2 a). In the cortex, fasudil hydrochloride induced a significant increase in CBF in the control, but not the saporin group compared to baseline (Fig. 2b). In addition, control mice that were administered fasudil hydrochloride had a significantly higher CBF compared to saporin-treated mice given the drug (Fig. 2b). These results suggest that denervated hippocampal vessels were still responsive to eNOS stimulation, while cortical vessels were not.

### eNOS protein expression in the hippocampus and cortex is differentially affected by saporin treatment

**Fig. 3:**
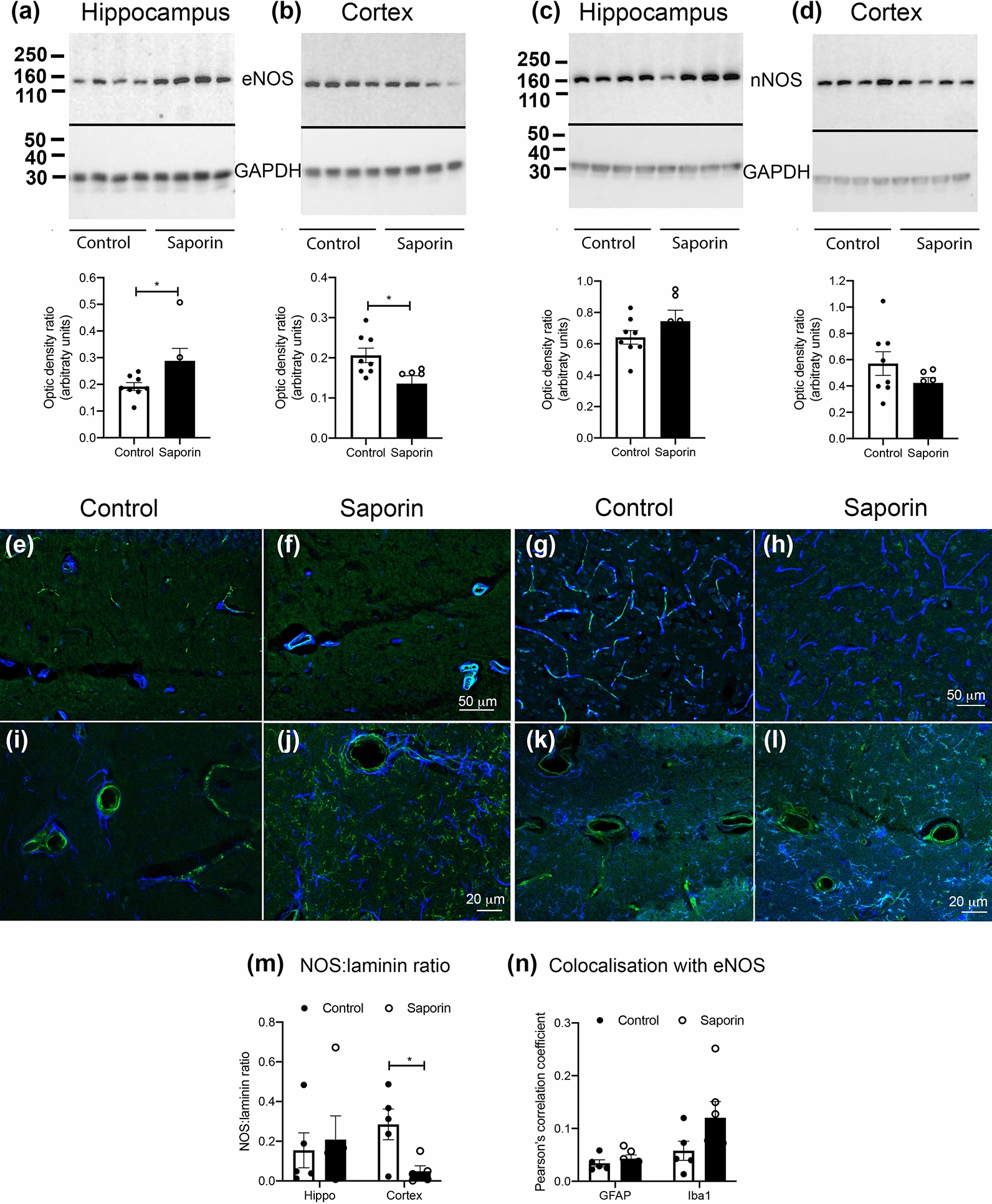
Regional variation in NOS expression and activity in control and saporin-treated mice. **a–d** Western blots and quantification of levels of eNOS (a and b) and nNOS (**c** and **d**) in the hippocampus (**a**, **c**) and cortex (**b**, **d**) of control and saporin-treated mice (n = 6–8/group). Molecular weight markers (kDa) are shown on the right hand side. The black line demarcates the original blot (upper) and the same blot re-probed for loading control (lower). **e–h** Photomicrographs showing the expression of total NOS (green) in laminin-positive vessels (blue) in the hippocampus (**e** and **f**) and cortex (**g** and **h**) of control (**e** and **g**) and saporin-treated mice (**f** and **h**). Note the stable expression of NOS in hippocampal vessels of saporin-treated mice, while NOS expression is significantly reduced in cortical vessels of saporin animals. **i–l** eNOS expression (green) in GFAP-positive astrocytes (blue, **i** and **j**) and Iba1-positive microglia (blue, **k** and **l**) in the hippocampus of control (**i** and **k**) and saporin mice (**j** and **l**). Colocalization between eNOS and GFAP or Iba-1 is shown as white-turquoise. **m** and **n** Quantification of NOS expression in blood vessels as a ratio to overall vessel density (**m**) and degree of colocalisation between eNOS and GFAP or Iba1 as measured by the Pearson’s correlation coefficient (**n**). n = 5/group. Scale bars for f and h = 50 μm; j and l = 20 μm. *p < 0.05

To determine whether regional differences in the responsiveness to fasudil hydrochloride were due to differences in the levels of NOS expression, cortical and hippocampal tissues were assessed by Western blotting using eNOS and nNOS-specific antibodies. eNOS expression was significantly higher in the hippocampus of saporin-treated mice compared to controls (Fig. 3a), while no statistically significant differences were observed between control and saporin mice in the cortex (Fig. 3b). Levels of nNOS did not differ significantly between control and saporin-treated mice in either the hippocampus or cortex (Fig. 3c, d). To determine if NOS expression may have been influenced by possible differences in vessel densities, the vascular expression of NOS in laminin-positive vessels was quantified in control and saporin tissues (Fig. 3e–h). Quantification of the NOS-to-laminin ratio confirmed the significant decrease in NOS expression in the cortex of saporin-treated mice (Fig. 3m). However, the NOS ratio in the hippocampus did not differ significantly between control and saporin mice (Fig. 3m). We also observed some NOS expression in glial cells in the hippocampus of saporin-treated mice. To determine if the increased eNOS expression detected by Western blot was due to expression in glial cells, hippocampal sections were stained with anti-eNOS and anti-GFAP or anti-Iba1 (Fig. 3i–l). These results confirmed minimal expression of eNOS in astrocytes, but some colocalization of eNOS with Iba1-positive microglia, which was higher in saporin-treated mice, although this did not reach statistical significance (*p* = 0.13, Fig. 3n). These results suggest that levels of eNOS are downregulated in the cortex, and upregulated in the hippocampus of saporin-treated mice and that increased eNOS expression in the hippocampus may be due in part to upregulation by microglia.

### Administration of fasudil hydrochloride increases IPAD in the hippocampus but not cortex of denervated mice

**Fig. 4:**
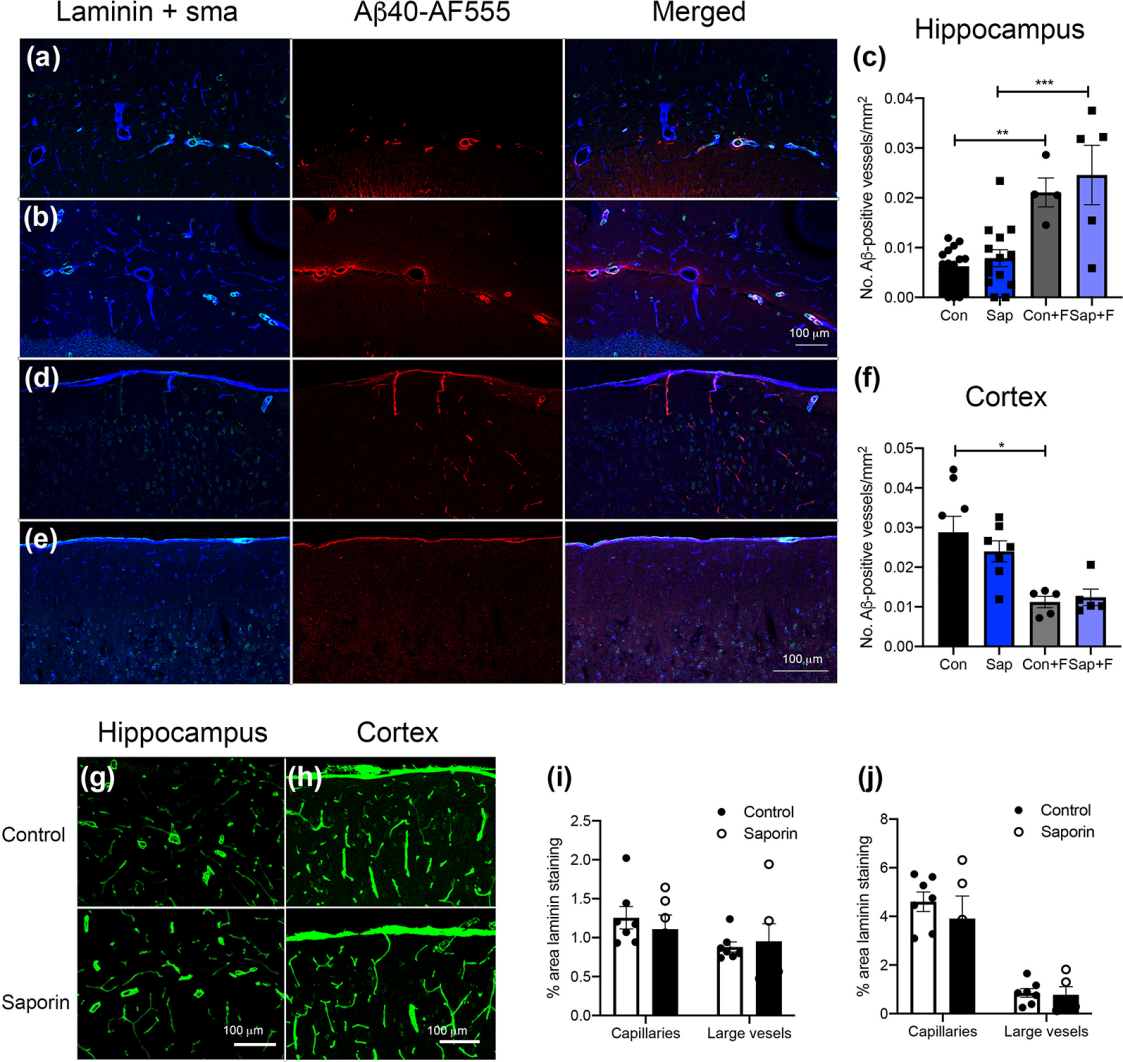
Administration of fasudil hydrochloride increases IPAD in the hippocampus, but not cortex of denervated mice. **a–f** Photomicrographs showing the distribution of human Aβ40-AF555 (red) at 5 min post-injection into the hippocampus (**a** and **b**) and at 2.5 min after injection into the cortex (**d** and **e**) of control mice at baseline (**a** and **d**) and after administration of fasudil hydrochloride (**b** and **e**). The cerebrovascular basement membrane was labelled with anti-laminin (blue) and smooth muscle cells were identified with anti-α smooth muscle actin (green). Quantification of the total number of Aβ40-containing vessels in the hippocampus (**c**) and cortex (**f**) of control (con) and saporin (sap)-treated mice at baseline (n = 14–16 for hippocampus and n = 7–8 for cortex) and after fasudil hydrochloride (+F) (n = 5/group for both regions). **g** and **h** Photomicrographs of laminin staining in the hippocampus (**g**) and cortex (h) of control (upper panels) and saporin-treated mice (lower panels). **i** and **j** Quantification of % area covered by laminin in the hippocampus (**i**) and cortex (**j**) of control and saporin animals. n = 5–7/group. Scale bars = 100 μm. *p < 0.05, **p < 0.01, ***p < 0.001

Our previous work has shown that saporin treatment significantly decreases cholinergic innervation of arterial smooth muscle cells in the hippocampus and cortex [61]. In vitro modelling supports the hypothesis that the localised arterial pulsations that regulate CBF [32] also provide the principle driving force for solute clearance from the brain via IPAD [1]. To determine if loss of cholinergic innervation altered IPAD, the pattern of distribution of human Aβ40-AF555 was evaluated following injection into the hippocampus or cortex of control and saporin-treated C57Bl/6 mice under physiologic and stimulated conditions. Triple-labelling immunohistochemistry demonstrated the presence of Aβ40-AF555 primarily in capillaries and arteries in both the hippocampus and cortex (Fig. 4a–f). Quantification of the number of hippocampal blood vessels that contained Aβ showed no difference between control and saporin-treated mice under baseline physiological conditions (Fig. 4c). Administration of fasudil hydrochloride resulted in significantly more Aβ-positive blood vessels in both control and saporin animals compared to baseline (Fig. 4b, c). However, fasudil treatment did not affect hippocampal vessel counts between control vs. saporin mice (Fig. 4c).

Preliminary assessment of IPAD in the cortex using the same parameters as those in the hippocampus (e.g. 0.5 μL Aβ40-AF555 + 5 min clearance) revealed a much smaller bolus of Aβ at the site of injection and very few Aβ-positive vessels were visible at 400 µm away from the injection site compared to the hippocampus (Additional file 4: Fig. 3a and b). Following a series of modifications (Additional file 4: Fig. 3c–e), the injection protocol for cortical injections was adapted to 0.25 µL Aβ40-AF555 with 2.5 min post-injection time (Fig. 4d–f), to allow for sufficient numbers of Aβ40-positive vessels to be counted. Similarly to the hippocampus, quantification of cortical vessels that contained Aβ revealed no baseline differences between control and saporin-treated mice (Fig. 4f). In control animals, administration of fasudil hydrochloride resulted in significantly fewer Aβ40-containing vessels (Fig. 4d–f). Although a similar trend was observed in saporin animals, the difference was not statistically significant (*p* = 0.08) and no difference was observed between control + fasudil and saporin + fasudil groups (Fig. 4f).

To determine if IPAD may have been influenced by differences in vessel number and/or microglia and astrocyte activation, densities of each were quantified in control and saporin-treated mice. The density of laminin-positive macrovessels and capillaries did not differ between control and saporin-treated mice in either the cortex or hippocampus (Fig. 4g–j), although capillary density was significantly higher in the cortex than the hippocampus in both treatment groups (*p* = 0.003). Similarly, quantification of Iba1 and GFAP staining revealed no effect of saporin treatment on astrocyte or microglial coverage of the hippocampus or cortex (Additional file 4: Fig. 3f–k).

These findings confirm that IPAD was not affected by differences in vessel density or glial activation and indicate that clearance of Aβ was stimulated by fasudil hydrochloride and that this responsiveness remains intact in the hippocampus, but not the cortex of denervated mice.

### Loss of cholinergic innervation increases CAA in the hippocampus of TetO-APP mice

**Fig. 5:**
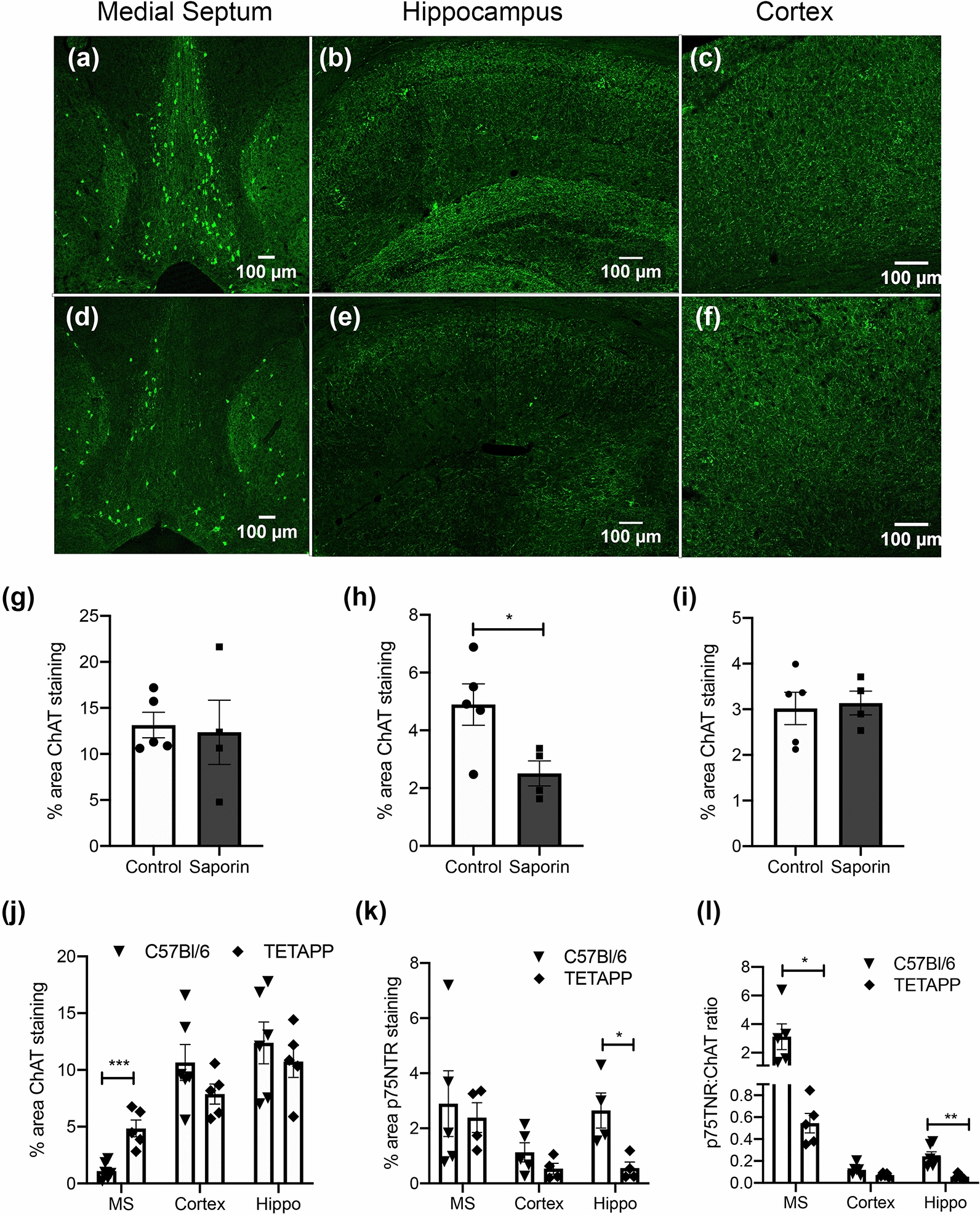
Distribution and quantification of cholinergic and p75NTR-positive neurons in control and saporin-treated TetO-APPSweInd mice. **a–f** Photomicrographs of ChAT staining in the medial septum and Diagonal band of Broca (**a** and **d**), hippocampus (**b** and **e**) and cortex (**c** and **f**) in control (**a**–**c**) and mu-saporin treated (**d**–**f**) TetO-APPSweInd mice. (**g–i**), Quantification of % area covered by ChAT-positive neurons in the medial septum (**g**) and fibres in the hippocampus (**h**) and cortex (**i**), n = 4–5/group. **j–l** Quantification of % area covered by ChAT (**j**) and p75NTR-positive (**k**) neurons and fibres and the ratio of ChAT:p75NTR expression (**l**) in the medial septum (MS), hippocampus (Hippo) and cortex of C57BL/6 and TetO-APPSweInd (TETAPP) mice, n = 5/group/strain. Scale bar = 100 μm. *p < 0.05, **p < 0.01,* **p < 0.001

To evaluate if cholinergic denervation potentiated Aβ pathology, 4-month old TetO-APPSweInd mice were administered saline or mu-saporin. Unexpectedly and in contrast to the observations made in the C57BL/6 mice, no significant differences were noted in the number of ChAT-positive neurons in the medial septum between control and saporin-treated mice (Fig. 5a, d, g). Significantly fewer cholinergic fibres were observed in the hippocampus of saporin vs control mice (Fig. 5b, e, h), while ChAT fibre density in the cortex was also unaffected by saporin treatment (Fig. 5c, f, i). To determine if the attenuated effect of saporin in the TetO-APPSweInd mice was due to endogenous differences in ChAT and p75NTR expression, fibre appearance and density was compared between TetO-APPSweInd mice and wildtype littermates. The morphology of fibres in the TetO-APPSweInd appeared dystrophic, with swollen varicosities and shorter processes than that of cholinergic fibres in the wildtype mice (Figs. 1b, c, 5b, c). The density of ChAT-positive neurons in the MS was significantly higher in TetO-APPSweInd mice compared to wildtype animals, although no differences in hippocampal or cortical ChAT fibre density were observed between strains (Fig. 5j). Analysis of p75NTR expression showed significantly lower receptor expression in the hippocampus of TetO-APPSweInd mice compared to wildtypes, while no differences were observed in the cortex or MS (Fig. 5k). Additional analysis found that the ratio of p75NTR to ChAT expression was significantly lower in the MS and hippocampus of TetO-APPSweInd mice compared to wildtype animals (Fig. 5l).

**Fig. 6:**
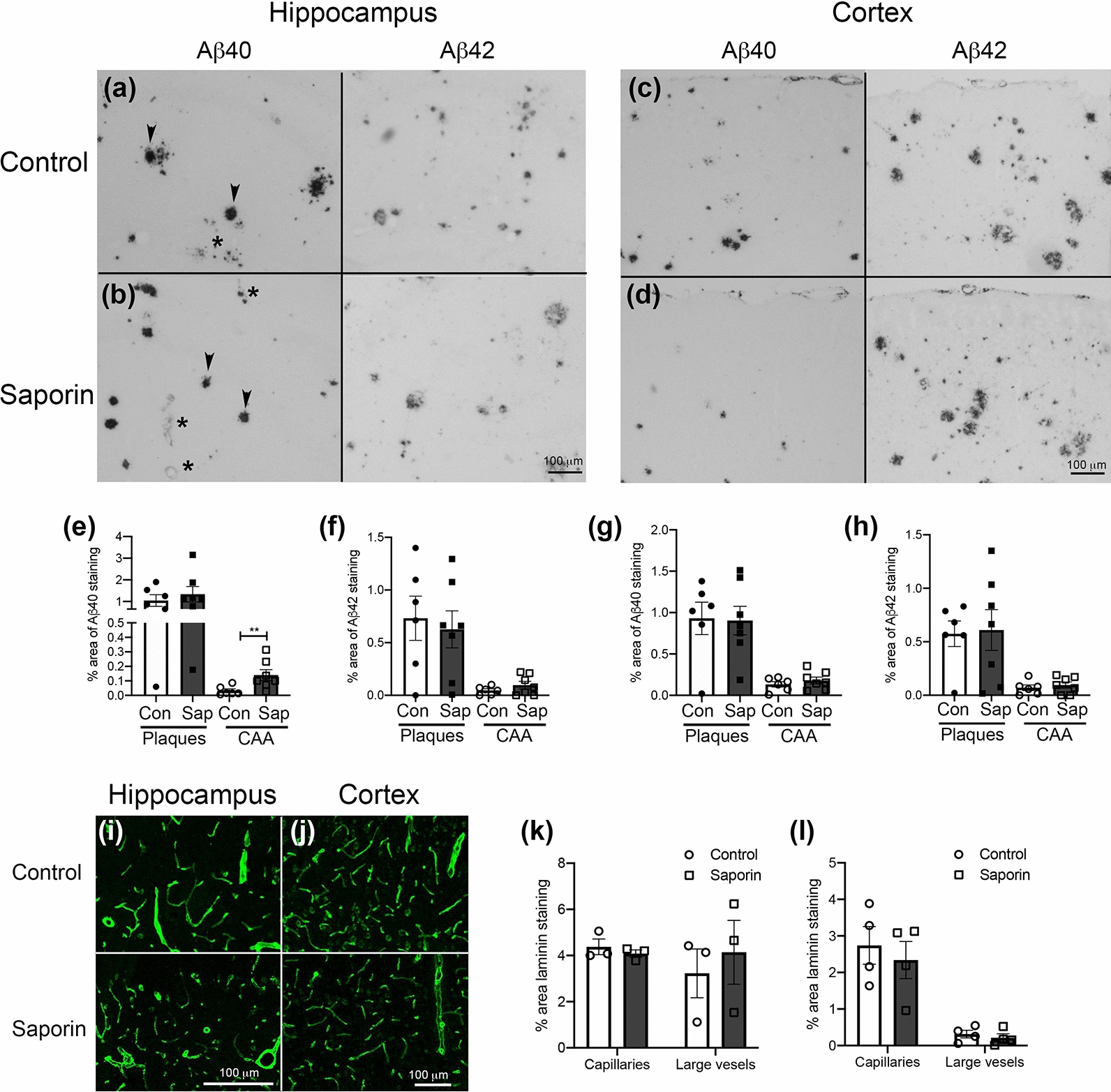
Loss of cholinergic innervation selectively increases Aβ40-positive CAA in the hippocampus of TetO-APP mice. **a–h** Photomicrographs of hippocampal (**a** and **b**) and cortical tissues (c and d) of TetO-APP mice stained with antibodies against human Aβ40 (**a**–**d**, left panels) and Aβ42 staining (**a**–**d**, right panels). Tissues from control animals are shown in the upper panels and saporin-treated tissues are shown in the lower panels. Arrowheads show plaques and asterisks show CAA-positive vessels. **e–h** Quantification of % area covered by Aβ40 (**e** and **g**) and Aβ42-positive (f and h) plaques and blood vessels in the hippocampus (**e** and **f**) and cortex (**g** and **h**) of TetO-APPSweInd mice, n = 6–7/group. **i–l** Photomicrographs of laminin staining in the hippocampus (**i**) and cortex (**j**) of control (upper panels) and saporin-treated mice (lower panels). **k** Histogram showing quantification of laminin density in control and saporin-treated mice. n = 3–5/group. Scale bars = 100 μm. **p < 0.01

Quantification of Aβ pathology in the hippocampus after saporin treatment showed no difference in the percentage area covered by Aβ40-positive plaques between control and saporin mice (Fig. 6a, b, e). However, Aβ40 CAA load was significantly higher in the saporin-treated mice (Fig. 6a, b, e). A similar but non-significant pattern of vascular Aβ42 staining was observed between control and saporin mice, while parenchymal Aβ42 was not affected by saporin treatment (Fig. 6a, b, f). In the cortex, no differences were observed between control and saporin-treated mice in the density of Aβ40-positive plaques or CAA (Fig. 6c, d, g). Likewise, the density of cortical parenchymal and vascular Aβ42 was unaffected by saporin treatment (Fig. 6c, d, h).

As with the C57BL/6 mice, vessel density between control and saporin-treated TetO-APPSweInd mice was similar in both the cortex and hippocampus (Fig. 6i–l). Analysis of GFAP and Iba1 expression revealed no significant difference in area coverage between treatment groups in either brain region (Additional file 4: Fig. 3l–q). These findings confirm that saporin administration did not significantly alter vessel density or glial activation in the TetO-APP mice and support a role for loss of cholinergic innervation in potentiating CAA pathology.

## Discussion

Results from this study suggest that loss of cholinergic innervation differentially affects cortical and hippocampal responsiveness to eNOS-stimulated increases in CBF and IPAD in wildtype mice, with hippocampal, but not cortical, vessels remaining responsive to stimulation. The death of cholinergic nerve fibres resulted in a significant and selective increase in Aβ40-positive CAA in the TetO-APPSweInd model of AD. These findings support the importance of the interrelationship between cholinergic innervation and vascular function in the aetiology and/or progression of CAA and suggest that regional vulnerability or resilience to loss of cholinergic dysfunction may contribute to the topographical nature of CAA (Additional file 4: Fig. 3).

Degeneration of cholinergic neurons and shrinkage of the basal forebrain are early features of AD and are associated with increased Aβ pathology, altered CBF and cognitive impairment [9, 22, 26, 82, 86]. In agreement with previous studies [58, 61], we found that intracerebral administration of mu-saporin, which selectively targets p75NTR-expressing neurons, caused the death of ChAT-positive neurons in the MS as well as their fibre projections in the cortex and hippocampus of wildtype mice.

ACh has a well-known vasodilatory effect in the brain and stimulation of the basal forebrain leads to increased cortical CBF [33, 35]. This effect is predominantly observed during NVC when release of ACh stimulates the production of NO via activation of eNOS or indirectly by stimulation of nNOS-containing interneurons [53]. Although ASL MRI can be used to measure NVC in the cortex using whisker or forepaw stimulation [49], to our knowledge similar methods are not available to stimulate NVC in the hippocampus of anesthetised animals. Therefore, to evaluate the impact of loss of cholinergic innervation on evoked CBF, we mimicked ACh activation of eNOS by using the selective ROCK inhibitor fasudil hydrochloride, which has been shown to increase CBF by stimulating the PI3K/Akt/eNOS pathway [68, 76]. Consistent with previous reports [50], loss of cholinergic innervation in the cortex and hippocampus did not affect baseline CBF in either region, supporting a primary role of ACh on CBF during NVC. However, while administration of fasudil hydrochloride was not able to evoke a change in CBF in the cortex of saporin-treated mice, denervated vessels in the hippocampus remained responsive to stimulation.

Because multiple downstream signalling pathways in addition to eNOS are regulated by ROCK activity, including those relating to smooth muscle contraction [45], we cannot definitively conclude that the observed effects were due to stimulation of eNOS. However, the findings that levels of eNOS were significantly decreased in the cortex and increased in the hippocampus of saporin-treated mice, support the hypothesis that loss of cholinergic innervation resulted in opposing effects on eNOS expression that aligned with the CBF response. Although eNOS is principally expressed by endothelial cells, previous studies have reported its expression in neurons, astrocytes and in microglia across various species [16, 75, 92]. Our observation that eNOS was expressed not only in blood vessels but also by microglia in the hippocampus of saporin-treated mice, suggests that the functional effects of fasudil hydrochloride in the hippocampus may also be due in part to activation of non-vascular cells. Although it is not clear why the effect of saporin treatment induced an opposite expression of eNOS between the cortex and hippocampus, endogenous NOS activity in both the nNOS- and eNOS-enriched fractions has previously been reported to be higher in the hippocampus compared to the cortex [74]. This is supported by previous reports showing that changes in CBF in the hippocampus were more proportional to changes in nNOS activity than in the cortex [52] and that the cortex is more sensitive than the hippocampus to inhibition of nNOS activity [43].

Previous studies have suggested that contractions of arterial smooth muscle cells are required for drainage of fluid along cerebral blood vessels [1, 3, 37], although whether this pulsation is sufficient to drive bulk flow of ISF and CSF remains controversial [11, 31]. Several studies have shown that vasoreactivity in AD is improved following treatment with AChEIs [71]. We hypothesised that there is a direct relationship between vasoreactivity and the efficiency of IPAD and that loss of cholinergic innervation would impair IPAD of Aβ in a similar pattern to that observed for CBF. No differences in IPAD were observed between control and saporin-treated mice in either brain region under baseline physiological conditions. Our observation that fewer Aβ-positive vessels were visible in the cortex after a 5 min diffusion period compared to the hippocampus, suggests that IPAD of Aβ may be endogenously faster in the cortex than in the hippocampus. We have previously reported differences in the efficiency of IPAD between subcortical brain regions that are differentially affected by CAA [27]. However, given the relatively small thickness of the mouse cortex [65], it is possible that the depth of injection into the cortex (0.5 mm from dura) may have flooded the subarachnoid space, even when using the smaller 0.25 µL volume. Therefore, more detailed in vivo tracer experiments are needed to clarify rates of IPAD between cortical and hippocampal regions. However, in agreement with other studies [21, 62], we also found that cerebrovascular density was significantly higher in the cortex compared to the hippocampus. This larger surface area may allow for solutes contained within the ISF to be more rapidly removed from the cortex than from the hippocampus under physiological conditions.

Administration of fasudil hydrochloride resulted in significantly more vessels with Aβ in the hippocampi of both control and saporin-treated mice, while in the cortex, fewer vessels were found to contain Aβ and this was observed in control mice only. Although the pattern of distribution was opposite between the two regions, we interpret both findings as representing increased IPAD at different rates of clearance. These findings are consistent with our CBF data and suggest that IPAD is significantly increased in the presence of vasodilation, which is in agreement with reports of impaired solute clearance from the brain during hypoperfusion [3, 37]. However, the similarity in the number of labeled cortical vessels between control + fasudil and saporin + fasudil mice suggests that other factors are also contributing to Aβ clearance in denervated mice. Although blood pressure was not monitored in the current experiments, previous studies have shown that fasudil hydrochloride does not alter systolic blood pressure in normotensive rodents or humans [44, 51, 57, 59], suggesting that the observed effects were unlikely to be due to changes in peripheral blood pressure. In addition, no differences in vessel density or markers of microglia and astrocytes were observed between control and saporin mice in either brain region. Although our findings are consistent with reports of an association between decreased eNOS expression and increased CAA [6, 81], recent work has shown that NVC is mediated in part by arteriole caveolae independent of eNOS activation [17]. Further work is required to determine the factors that regulate Aβ clearance when cholinergic signalling is attenuated.

Previous studies have reported a relationship between basal forebrain degeneration and Aβ pathology in the cortex [26] and basal forebrain atrophy has been suggested to predict cortical Aβ burden [82]. Induced loss of cholinergic neurons in rodent models of AD has also been associated with increased Aβ plaque deposition [48, 67], however most studies have not specifically investigated the effect on vascular Aβ. In the present study, administration of mu-saporin in TetO-APPSweInd mice resulted in a loss of cholinergic neurons that was only significant in the hippocampus. The reasons for the discrepancies between the degree of loss between the C57BL/6 and TetO-APPSweInd mice are not clear, but may relate to the dystrophic appearance of cholinergic fibers and decreased p75NTR:ChAT ratio observed in the TetO-APPSweInd mice. As binding of the p75NTR by Aβ is known to induce apoptosis [95], it may be that pre-existing Aβ pathology caused damage to cholinergic neurons and fibres that induced a downregulation in p75NTR expression and decreased receptor availability for mu-saporin binding.

Although unexpected, the difference in sensitivity to saporin treatment between the cortex and hippocampus provided an internal control to study the effect of cholinergic loss on Aβ pathology. We found that loss of cholinergic innervation in the hippocampus was associated with a significant increase in Aβ40-positive vessels, consistent with the preferential deposition of Aβ40 in the vasculature in AD [30, 80]. By contrast, CAA load was not affected in the cortex where cholinergic fibre density was not altered by saporin treatment. Parenchymal plaque load did not differ between control and saporin-treated mice in either region. These findings are consistent with previous studies showing significantly more endogenous CAA in the absence of changes in parenchymal changes or changes in APP processing in rabbits administered saporin [10, 70]. These findings are also similar to a study which found that age-related loss of perivascular cholinergic innervation in the cortex did not significantly correlate with increased cortical plaque load in the Tg2576 AD mouse model [46]. However, other studies have reported increased plaque load and elevated concentrations of soluble Aβ following saporin-induced cholinergic loss in the APP/PS1 and Tg2576 mouse models [24, 48, 67]. Many factors may have contributed to these different observations, including the degree of cholinergic degeneration, age of the mice and amount of pre-existing Aβ pathology before saporin treatment, as well as the ratio of Aβ40:Aβ42 and progression of pathology between the different mouse models. Despite these discrepancies, our results support a consensus that loss of cholinergic innervation contributes to increased Aβ pathology.

In addition to the previously discussed limitations related to inducing NVC in the hippocampus of anesthetised animals and assessment of IPAD ex vivo, this study has several other weaknesses. The saporin model induces loss of basal forebrain cholinergic neurons in a retrograde manner [47], and over a more rapid timeframe than that observed in AD, which may activate a strong acute inflammatory reaction and/or the development of compensatory mechanisms in the animal model that are not present in AD. In addition, as age is the major risk factor for both sporadic AD and CAA, additional experiments are needed to determine whether the effects of cholinergic denervation on CBF and IPAD in the cortex and hippocampus seen here in young adult mice are also observed in aged animals.

## Conclusions

Despite these limitations, findings from this study support a role for loss of cholinergic innervation in the aetiology and/or progression of CAA and suggest that this may be related to eNOS-mediated vasodynamics that contribute to clearance of Aβ from the brain via IPAD pathways. Therefore, combined targeting of eNOS and cholinergic signalling/activation may represent a new mechanism to improve the efficiency of Aβ removal and reduce its deposition as CAA.

## Supplementary Information

**Additional file 1**: **Table 1** List of source of primary and secondary antibodies used for immunohistochemistry.

**Additional file 2**: **Fig. 1 a–d** Photomicrographs of diffuse parenchymal plaques identified by the anti-Aβ40 antibody (**a**) and senile plaques stained by the anti-Aβ42 antibody (**b**) in TetO-APPSweInd mice. No staining was observed after pre-absorption of the Aβ40 antibody with Aβ40 peptide (1:10 molar ratio, **c**). Sections incubated after pre-absorption of Aβ40 with anti-Aβ40 + anti-Aβ42 (**d**) showed a similar pattern of staining to that of sections incubated with the anti-Aβ42 antibody alone. **e–h** No staining was observed in tissue sections from C57Bl/6 mice incubated with fluorescently-conjugated secondary antibodies alone.

**Additional file 3: Fig. 2 a–f** Photomicrographs showing expression of ChAT (green), p75NTR (red) and their colocalization (yellow) in neurons in the medial septum (**a** and **b**) and fibers in the hippocampus (**c** and **d**) and cortex (**e** and **f**) of C57Bl/6 mice. Animals received an intracerebroventricular injection of either PBS (control, **a**, **c** and **e**) or mu-saporin (**b**, **d** and **f**). Saporin treatment significantly reduced expression of p75NTR, ChAT-positive cell bodies and fibers in the medial septum (**b**), hippocampus (**d**) and cortex (**f**). Images of the hippocampus are composed of individual overlapping images stitched together using Fiji. Scale bars: a, b, e, f = 250 μm; c and d = 100 μm.

**Additional file 4: Fig. 3 a** and **b** Photomicrographs showing the distribution of 0.5 μL human Aβ40-AF555 (red) at 400 μm away from the injection site after 5 min post-injection (PI) into the hippocampus (**a**) and cortex (**b**) of control mice. The cerebrovascular basement membrane was labelled with anti-laminin (blue) and smooth muscle cells were identified with anti-α smooth muscle actin (green). **c**–**e** Photomicrographs showing the distribution of Aβ40-AF488 (green) in the cortex of C57BL/6 mice, at 400 μm away from the injection site. The volume and post-injection (PI) time is indicated for 3 combinations that were tested to determine the optimal parameters for quantification of Aβ-positive vessels. **f**–**k** Photomicrographs and quantification of GFAP (**f**, **g**, **j**) and Iba1 (**h**, **i**, **k**) staining in the hippocampus (**f**, **h**) and cortex (**g**, **i**) of control (con, upper panels) and saporin-treated C57Bl/6 mice (sap, lower panels). **l**–**q** Photomicrographs and quantification of GFAP (**l**, **m**, **p**) and Iba1 (**n**, **o**, **q**) staining in the hippocampus (**l**, **n**) and cortex (**m**, **o**) of control (con, upper panels) and saporin-treated TetO-APPSweInd mice (sap, lower panels). n = 3–5/group. Scale bars = 100 μm.

## Data Availability

The datasets used and/or analysed during the current study available from the corresponding author on reasonable request.

## Abbreviations

7-NI: 7-Nitroindazole
Aβ: Beta amyloid
ACh: Acetylcholine
AChEI: Acetylcholinesterase inhibitor
AD: Alzheimer’s disease
APP: Amyloid precursor protein
ASL: Arterial spin labelling
CAA: Cerebral amyloid angiopathy
CBF: Cerebral blood flow
CVBM: Cerebrovascular basement membranes
ChAT: Choline acetyltransferase
eNOS: Endothelial nitric oxide synthase
GAPDH: Glyceraldehyde 3-phosphate dehydrogenase
GFAP: Glial acidic fibrillary protein
Iba1: Ionized calcium binding adaptor molecule 1
ICV: Intracerebroventricular
IPAD: Intramural periarterial drainage
iNOS: Inducible nitric oxide synthase
ISF: Interstitial fluid
L-NAME: Nitro-l-arginine Methyl Ester Hydrochloride
MRI: Magnetic resonance imaging
MS: Medial septum
nNOS: Neuronal nitric oxide synthase
NVC: Neurovascular coupling
P75NTR: p75 neurotrophin receptor
ROCK: Rho- associated, coiled-coil containing protein kinase
