# Supplementary material for: Loss of cholinergic innervation differentially affects eNOS-mediated blood flow, drainage of Aβ and cerebral amyloid angiopathy in the cortex and hippocampus of adult mice
Source: Acta Neuropathol Commun. 2021 Jan 7;9:12. doi: 10.1186/s40478-020-01108-z (PMC7791879; doi:10.1186/s40478-020-01108-z)
Supplement: Supplementary file 1 — Additional file 1: Table 1 List of source of primary and secondary antibodies used for immunohistochemistry. [file 40478_2020_1108_MOESM1_ESM.docx]

**Supplemental Table 1** List of source of primary and secondary antibodies used for immunohistochemistry

| Antigen | Antibody host | Working dilution | Antigen retrieval | Source |
| --- | --- | --- | --- | --- |
| *Primary Antibodies* |  |  |  |  |
| Anti-Aβ_1-40_ | Rabbit | 1:100 | 70% Formic acid (v/v) (45 s at RT) | Merck Millipore (Dorset, UK)  Cat. AB5074P |
| Anti-Aβ_1-42_ | Rabbit | 1:100 | 70% Formic acid (v/v) (45 s at RT) | Merck Millipore  Cat. AB5078P |
| Anti-ChAT | Goat | 1:75 | - | Merck Millipore  Cat. AB144P |
| Anti-eNOS | Mouse | 1:200 |  | BD Biosciences  (Wokingham, UK)  Cat. 610296 |
| Anti-GFAP | Chicken | 1:500-1:2000 |  | Abcam (Cambridge, UK  Cat. ab4674 |
| Anti-Iba1 | Rabbit | 1:500 | 0.01 M Sodium Citrate 0.01% tween pH 6 (15 min at ~90⁰C) | Abcam  Cat. ab178846 |
| Anti-Laminin | Rabbit | 1:350 | 1 mg/ml Pepsin in 0.2 M HCl (30 s at 37⁰C) | Sigma-Aldrich (Dorset, UK)  Cat. L9393 |
| Anti-NOS | Mouse | 1:200 |  | Novus Biologicals  (Abingdon, UK)  Cat. NB120-2801 |
| Anti-p75NTR | Rabbit | 1:400 |  | Merck Millipore  Cat. 07-476 |
| Anti-SMA-FITC conjugated | Mouse | 1:350 | - | Sigma-Aldrich  Cat. F3777 |
|  |  |  |  |  |
| *Secondary antibodies* |  |  |  |  |
| Anti-chicken AlexaFluor®633 | Goat anti-chicken | 1:200 | - | Fisher Scientific UK  Cat. 10444562 |
| Anti-goat AlexaFluor®488 | Donkey anti-goat | 1:200 | - | Fisher Scientific UK  Cat. 10246392 |
| Anti-mouse AlexaFluor®488 | Goat anti-mouse | 1:200 | - | Fisher Scientific UK  Cat. 10696113 |
| Anti-rabbit  AlexaFluor®488 | Goat anti-rabbit | 1:200 | - | Fisher Scientific UK  Cat. 10696113 |
| Anti-rabbit  AlexaFluor®633 | Goat-anti-rabbit | 1:200 | - | Fisher Scientific UK  Cat. 10104192 |
