# Supplementary figures and images for: Loss of cholinergic innervation differentially affects eNOS-mediated blood flow, drainage of Aβ and cerebral amyloid angiopathy in the cortex and hippocampus of adult mice
Source: Acta Neuropathol Commun. 2021 Jan 7;9:12. doi: 10.1186/s40478-020-01108-z (PMC7791879; doi:10.1186/s40478-020-01108-z)

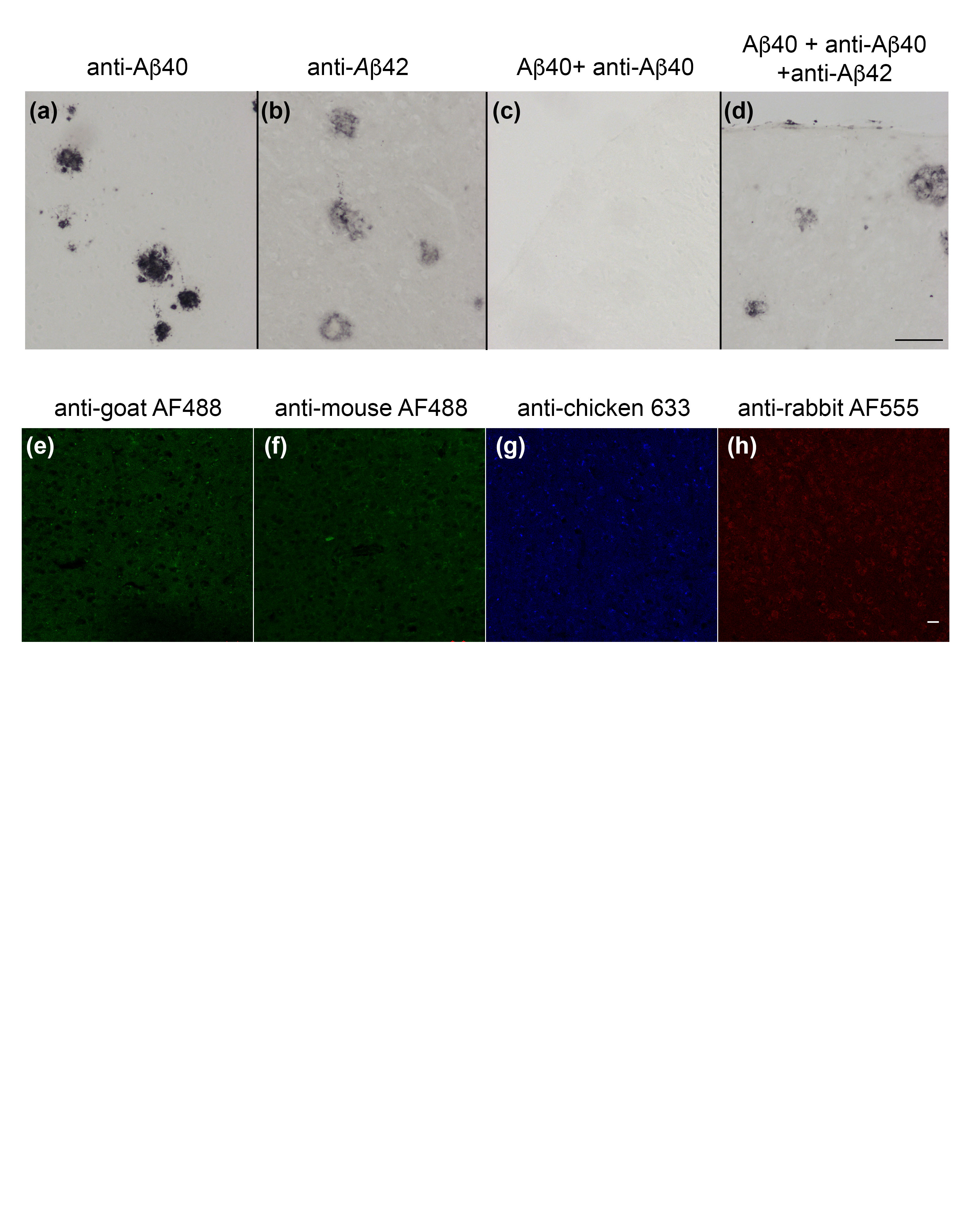

Supplement: Supplementary file 2 — Additional file 2: Fig. 1 a–d Photomicrographs of diffuse parenchymal plaques identified by the anti-Aβ40 antibody (a) and senile plaques stained by the anti-Aβ42 antibody (b) in TetO-APPSweInd mice. No staining was observed after pre-absorption of the Aβ40 antibody with Aβ40 peptide (1:10 molar ratio, c). Sections incubated after pre-absorption of Aβ40 with anti-Aβ40 + anti-Aβ42 (d) showed a similar pattern of staining to that of sections incubated with the anti-Aβ42 antibody alone. e–h No staining was observed in tissue sections from C57Bl/6 mice incubated with fluorescently-conjugated secondary antibodies alone. [file 40478_2020_1108_MOESM2_ESM.jpg]

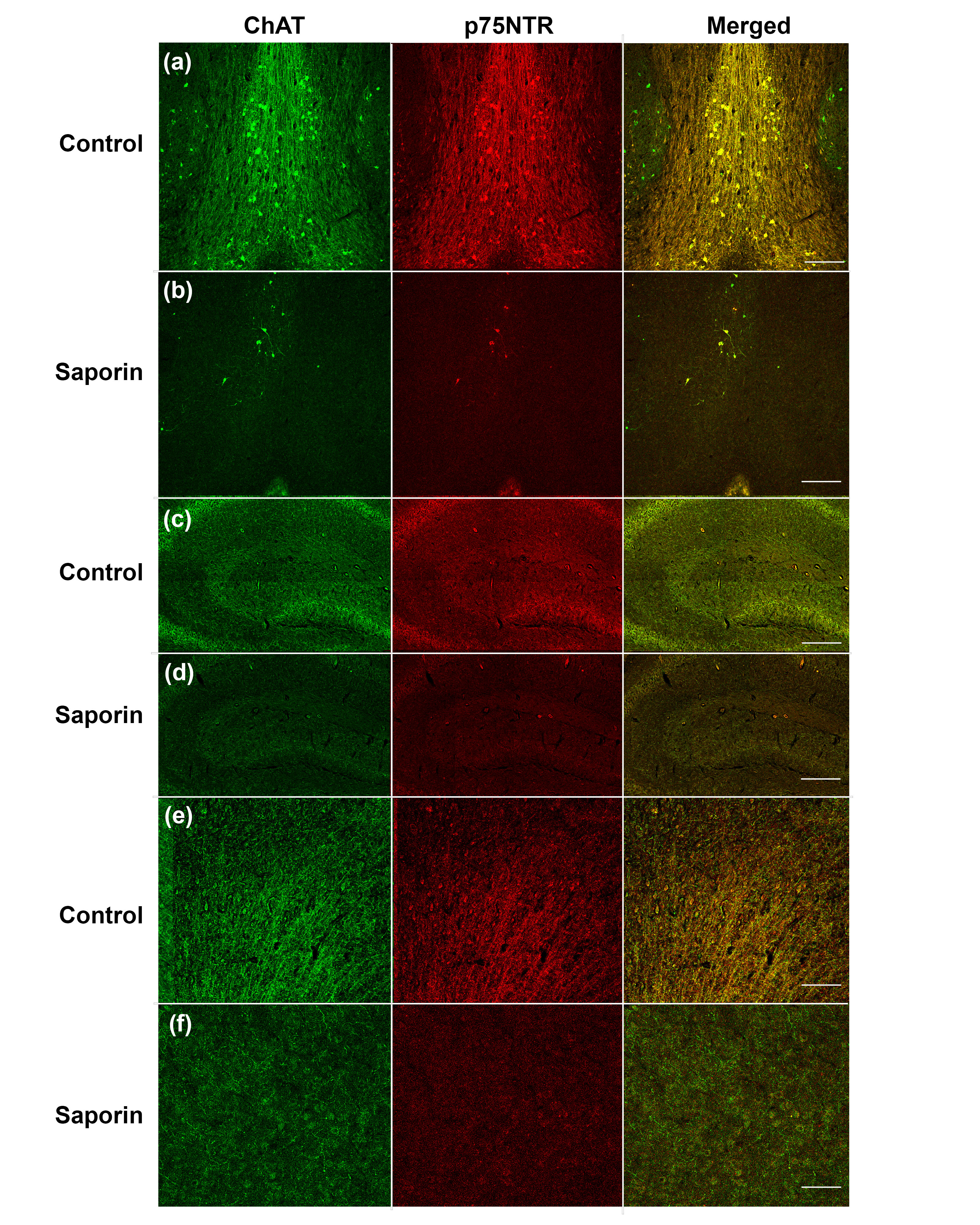

Supplement: Supplementary file 3 — Additional file 3: Fig. 2 a–f Photomicrographs showing expression of ChAT (green), p75NTR (red) and their colocalization (yellow) in neurons in the medial septum (a and b) and fibers in the hippocampus (c and d) and cortex (e and f) of C57Bl/6 mice. Animals received an intracerebroventricular injection of either PBS (control, a, c and e) or mu-saporin (b, d and f). Saporin treatment significantly reduced expression of p75NTR, ChAT-positive cell bodies and fibers in the medial septum (b), hippocampus (d) and cortex (f). Images of the hippocampus are composed of individual overlapping images stitched together using Fiji. Scale bars: a, b, e, f = 250 μm; c and d = 100 μm. [file 40478_2020_1108_MOESM3_ESM.jpg]

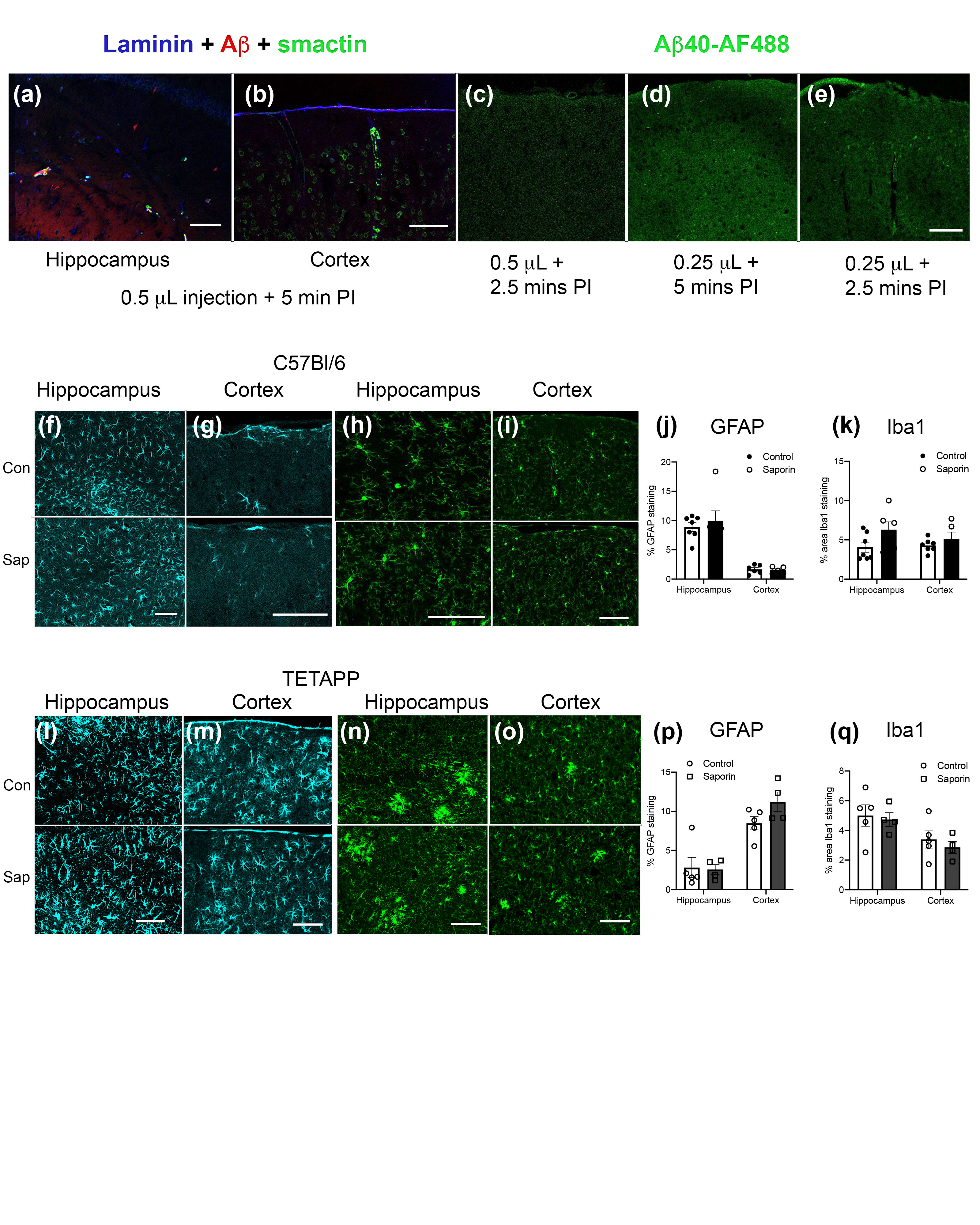

Supplement: Supplementary file 4 — Additional file 4: Fig. 3 a and b Photomicrographs showing the distribution of 0.5 μL human Aβ40-AF555 (red) at 400 μm away from the injection site after 5 min post-injection (PI) into the hippocampus (a) and cortex (b) of control mice. The cerebrovascular basement membrane was labelled with anti-laminin (blue) and smooth muscle cells were identified with anti-α smooth muscle actin (green). c–e Photomicrographs showing the distribution of Aβ40-AF488 (green) in the cortex of C57BL/6 mice, at 400 μm away from the injection site. The volume and post-injection (PI) time is indicated for 3 combinations that were tested to determine the optimal parameters for quantification of Aβ-positive vessels. f–k Photomicrographs and quantification of GFAP (f, g, j) and Iba1 (h, i, k) staining in the hippocampus (f, h) and cortex (g, i) of control (con, upper panels) and saporin-treated C57Bl/6 mice (sap, lower panels). l–q Photomicrographs and quantification of GFAP (l, m, p) and Iba1 (n, o, q) staining in the hippocampus (l, n) and cortex (m, o) of control (con, upper panels) and saporin-treated TetO-APPSweInd mice (sap, lower panels). n = 3–5/group. Scale bars = 100 μm. [file 40478_2020_1108_MOESM4_ESM.jpg]
